# Nanoscale analysis of frozen honey by atom probe tomography

**DOI:** 10.1038/s41598-022-22717-9

**Published:** 2022-10-22

**Authors:** Tim M. Schwarz, Jonas Ott, Helena Solodenko, Guido Schmitz, Patrick Stender

**Affiliations:** Institute for Materials Science, Chair of Materials Physics, University of Stuttgart, Heisenbergstr. 3, 70569 Stuttgart, Germany

**Keywords:** Organic molecules in materials science, Structural properties

## Abstract

Three-dimensional reconstruction of the analysed volume is one of the main goals of atom probe tomography (APT) and can deliver nearly atomic resolution (~ 0.2 nm spatial resolution) and chemical information with a mass sensitivity down to the ppm range. Extending this technique to frozen biological systems would have an enormous impact on the structural analysis of biomolecules. In previous works, we have shown that it is possible to measure frozen liquids with APT. In this paper, we demonstrate the ability of APT to trace nanoscale precipitation in frozen natural honey. While the mass signals of the common sugar fragments C_*x*_H_*y*_ and C_*x*_O_*y*_H_*z*_ overlap with (H_2_O)_*n*_H from water, we achieved correct stoichiometric values via different interpretation approaches for the peaks and thus determined the water content reliably. Next, we use honey to investigate the spatial resolution capabilities as a step toward the measurement of biological molecules in solution in 3D with sub-nanometer resolution. This may take analytical techniques to a new level, since methods of chemical characterization for cryogenic samples, especially biological samples, are still limited.

## Introduction

Atom Probe tomography (APT)^1–6^ has become an advantageous tool to investigate chemical structures on the atomic scale^7^ with a mass sensitivity in the ppm range and a spatial resolution of ~ 0.2 nm. APT is well established for metals and ceramics and widely used for semi-conducting materials^8^ as well as “hard” organic matter such as teeth, chiton and bones^9–16^. One of the current trends in APT is to attain a deeper understanding of soft matter regarding evaporation and fragmentation behavior. Thin films of polyelectrolytes^17^ and self-assembled monolayers (SAMs)^18–20^ have already been examined for their chemical structure by APT. These measurements revealed that organic materials evaporate very differently from metals or semiconducting materials. Instead of individual atoms, the organic material evaporates in larger fragments or even as complete molecules, depending on the measurement conditions^21^. This evaporation behavior complicates peak identification due to mass peak overlap and leads to ambiguity in peak identification. Additionally, it limits spatial resolution. On the other hand, the measurement of larger molecular fragments enables the identification of structural entities of large biological molecules.

Nevertheless, the interest in exploring the chemical structure of biological materials on the nanometer length scale is enormous^22,23^. To the present day, most biological materials are not analyzed in their natural environment by APT, resulting in changes in the 3D constitution of the original structure. The molecules are measured so far in crystalline form^24^, embedded in resin^25^ or silica glass^26^, or attached via adhesion to the tip apex^27^. Several attempts have been made previously to measure thin layers of frozen water in high electric fields^28–32^. With the newly available cryo-transfer systems^33–36^ it is now possible to transfer the samples between different instruments, which provides access to liquid specimens and opens a new era in APT. Since this development, several measurements of frozen liquids were reported^21,37–44^. This may enable the analysis of large volumes of frozen liquids, providing statistically reliable information on the evaporation and fragmentation behavior of liquids. In future, it also opens the way for atomic studies of biological samples in their natural environment, as well as catalytic reactions at liquid/solid interfaces even allowing to study of wet corrosion mechanisms. For measurements of aqueous solutions or molecules/particles dissolved in an aqueous environment, there are still no normed standards regarding measurement conditions and reconstruction parameters available.

In a previous work^44^, we followed a bottom-up approach and first analyzed the pure water matrix (Milli-Q). That study focused on understanding the fragmentation and evaporation behavior and determined the fingerprint of typical water fragments. In a consequent second step, we analyzed a saturated glucose solution and compared it to bulk glucose and water to broaden the chain of evidence.

This demonstrated the potential that a 3D sub-nanometer resolution of dissolved biological molecules can be achieved by APT^43^. The mass spectra of water and a saturated glucose solution revealed a complex structure with different clusters of protonated molecules of varying sizes^43^. Initially, the significant overlap of the peaks, stemming from water or glucose, made the identification of the peaks deriving from combinations of H, C, and O, difficult. However, in the previous work^43^, we investigated this overlap and its possible consequences for the stoichiometric ratio by comparing the spectra of saturated glucose solution with those of pure water and pure solid bulk glucose. This allowed a re-identification by using protonated water clusters (H_2_O)_*n*_H^+^ as a clear fingerprint. With this knowledge, now natural honey, which is a supersaturated solution consisting of a mixture of different monosaccharides and higher sugars in water, can be investigated on the nanoscale.

Honey is a natural product made by honeybees from the nectar of plants, secretions of living parts of plants or excretions of plant-sucking insects^45,46^. With the help of enzymes (diastase, invertase and other substances) the nectar is modified by breaking larger saccharides, especially sucrose, into monosaccharides such as glucose and fructose^47^ and partial evaporation of the existing water. Therefore, honey is mainly composed of monosaccharides glucose/fructose, disaccharides (sucrose and maltose), higher sugars, enzymes, acids, vitamins, minerals, aromatic substances, and water^47,48^. Monosaccharides represent about 75% of the sugar content, 10–15% of disaccharides and the rest consists of higher sugars (trisaccharide and higher oligosaccharide). The monosaccharides glucose and fructose consist of a pyranose ring which is made of five carbon atoms and one oxygen atom, with a hydroxymethyl group at C5 for glucose and at C1 for fructose. Fructose can also exist in a furanose structure that consists of a ring of four carbon atoms and one oxygen atom but is completed with two hydroxymethyl groups at C1 and C4. However, the molecular formula for both monosaccharides is C_6_O_6_H_12_. As a disaccharide, sucrose consists of a glucose and a fructose molecule connected through an α-1,4 binding. Thus, the molecular formula for sucrose is C_12_O_11_H_22_. Honey can have variable compositions, depending on the botanical and geographical origin, type of bees, climate, processing, and storage^48^. The reported chemical composition of honey in different countries is listed in Table S1 in the Supplementary Information^48^. Typically, 830 g/L consists of sugars, which is higher than the individual saturation limit of glucose (470 g/L) and fructose (790 g/L) in water. The water content can vary between 10.2 and 20.47% (European)^48^, which is very small compared to the saturated glucose solution investigated earlier^43^ and thus resulting in a higher viscosity of honey. Glucose has a lower solubility in water than fructose and precipitates and crystallizes therefore more rapidly. The tendency of the crystallization of honey can be predicted by the glucose/fructose ratio, whereby a higher ratio leads to faster crystallization^49–51^. Another study used the glucose/water ratio as an indicator to predict crystallization^52^. Honey has hygroscopic characteristics and can absorb moisture from the environment.

In this work, we go a step further and study natural honey, a supersaturated solution, and demonstrate APT’s capabilities to measure nano-precipitates within frozen natural honey. While the mass signals of the abundant sugar fragments C_*x*_H_*y*_ and C_*x*_O_*y*_H_*z*_ overlap with (H_2_O)_*n*_H^+^ from water, we were able to obtain correct stoichiometric values by different interpretation approaches for the peaks and thus reliably determine the water content. Next, we investigated the spatial resolution capabilities using honey as a step toward measuring biological molecules in solution in 3D with sub-nanometer resolution.

## Results

For the APT analysis, frozen honey tips were prepared as described under “Experimental section/methods” (see below). We determined suitable analysis conditions where the number of multi-hit events was kept to a minimum and continuous evaporation was achieved. These conditions were obtained using UV laser pulses at a repetition rate of 100 kHz and laser energies between 40 and 80 nJ per pulse at a base temperature of 60 K. Measurements were performed with a detection rate of 300–400 atoms per second (0.003 atoms per pulse) yielding reasonably homogeneous detector patterns and a steady, continuously increasing voltage over time (see Supplementary Information Fig. S1). In total, three successful data sets were obtained.

**Figure 1:**
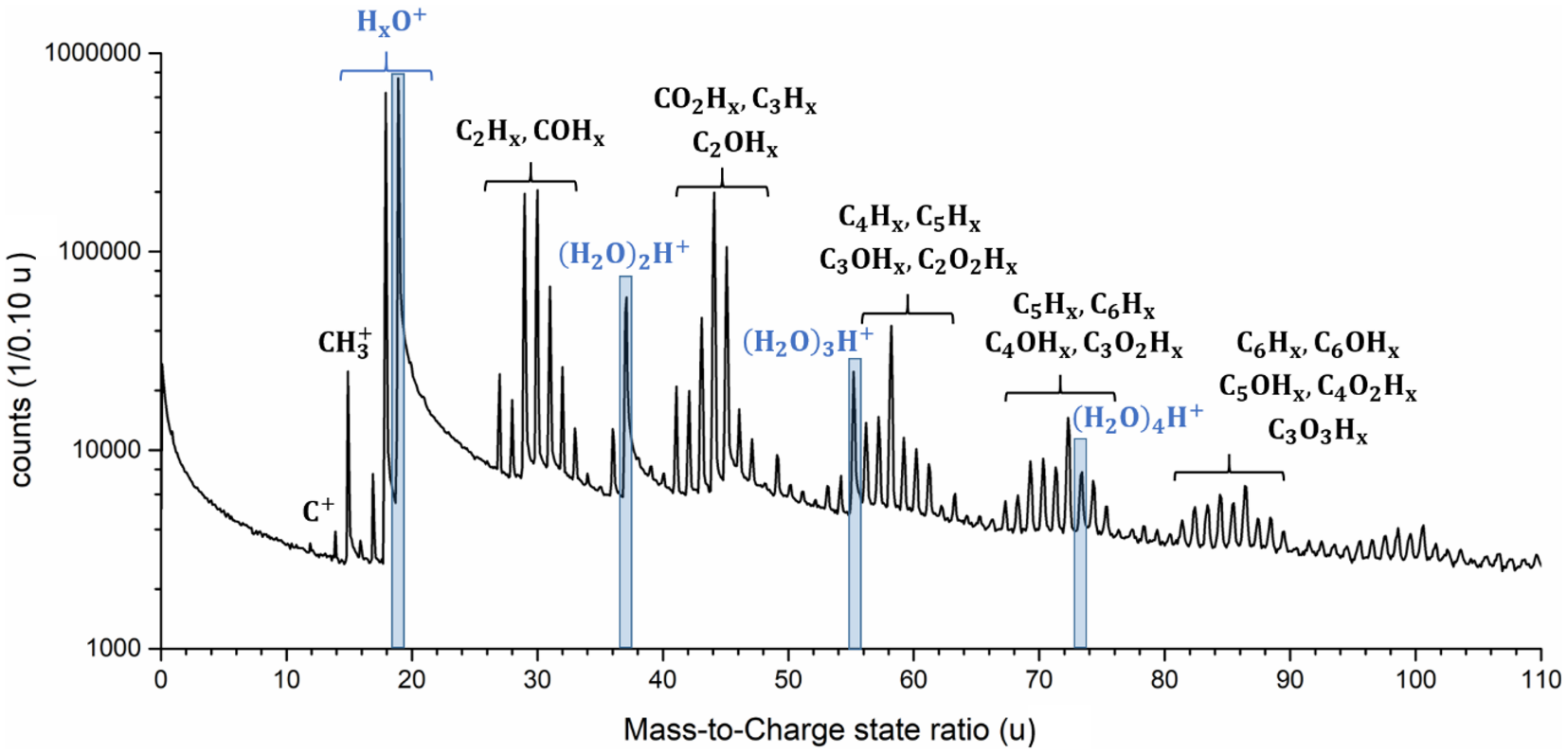
APT mass spectrum of honey with a mass-to-charge state ratio from 0 to 110 u as a logarithmic plot. The protonated water molecules/clusters with the molecular formula (H_2_O)_*n*_H^+^ with *n* = 1–4 are highlighted. Different groups of peaks are marked.

The typical atom probe mass spectrum of natural honey (Fig. 1) shows a similar complex fragmentation behavior as in our previous measurements of water, glucose solution and solid bulk glucose^43,44^. The number of individual mass signals increased, whereof about 100 peaks can be identified. 60 million events in total were detected for the shown spectra. The first 10 million atoms were discarded to avoid misinterpretation from residues on the surface due to the preparation process.

Peak identification becomes ambiguous due to many possible combinations of charge states and chemical species. The lowest possible charge states are assumed in assigning a molecular identity, since higher charge states are unlikely due to the low observed evaporation field of water or glucose solution^28,29,53^. The likely possibilities for peak assignment are listed in Table S2 (see Supplementary Material).

As a general guideline, measurement conditions should suppress multiple hit events since they complicate data analysis. If the time and/or spatial distance is too small, some of the multiple events will not be detected, which biases the compositional analysis and therefore the reconstruction of structures. Such multiple events occur when fragments co-evaporate from the surface. Another possibility, which is more interesting in the case of peak interpretation, is the further dissociation of molecular species during acceleration after field desorption. Therefore, correlation plots may become a useful tool to verify whether molecular dissociations or co-evaporation of species occur^54^ and provide a better understanding of the evaporation process.

Under the chosen measurement conditions, single hit events represent the majority of the measurement with 83.73%, followed by 8.92% double and 4.2% triple hit events. Figure S4 (see Supplementary Material) shows the correlation plot of the honey measurement. No dissociation tracks were observed, in contrast to the earlier pure water measurement^44^. No significant difference in the occurring species could be observed between the separated spectra of the single, double, triple… events (see Supplementary Material, Figs. S2–S4).

### Comparison of honey to water, glucose solution and glucose bulk

Water has been the primary component in the case of the earlier measured glucose solution. The ionization field strength of water (2–10 V/nm)^28,29,53^ is rather low in comparison to conventional metallic samples. In contrast, the main component of honey is made of monosaccharides (glucose, fructose) and higher bonded sugars, while only a minority fraction of 12–22 at.% is water. The exact evaporation fields of glucose and other sugars are still unknown, but we do expect them to be significantly higher than that of water.

**Figure 2:**
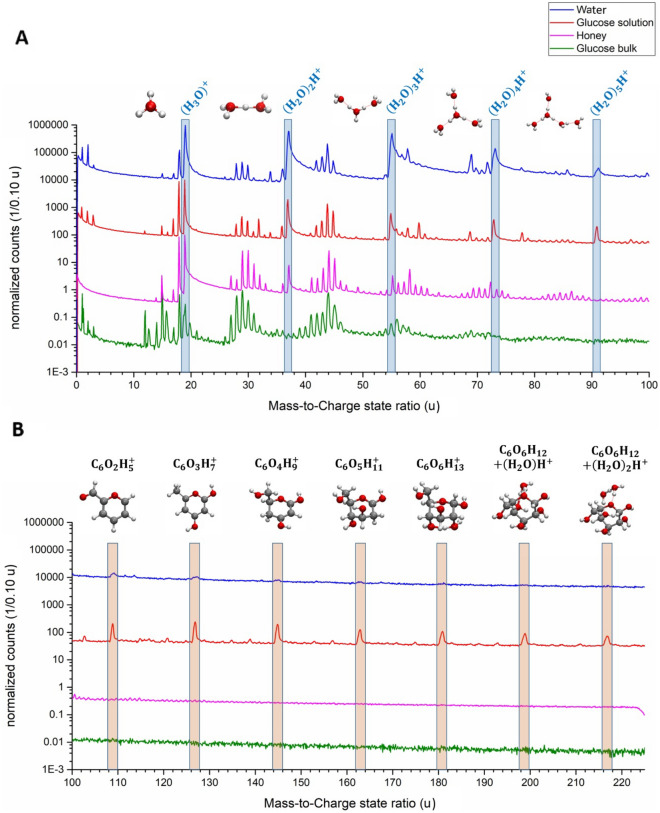
Comparison of the mass spectra of pure water (blue), a saturated glucose solution (red), glucose as bulk material (green) and honey (purple). All mass spectra are normalized to the respective highest peak from the mass-to-charge state ratio of m/q = 0–110 u as a logarithmic plot. The water spectra were shifted upwards by a factor of 10^6^, glucose solution by 10^4^ and honey by a factor of 10^2^ to improve the visibility of the mass spectra. In (**A**) The protonated water molecules/clusters are highlighted and the respective molecular structure of (H_2_O)_*n*_H^+^ with *n* = 1–5 is shown. In (**B**) the peaks stemming from glucose from m/q = 91 up to 217 u are marked and the respective molecular structures are shown.

**Figure 3:**
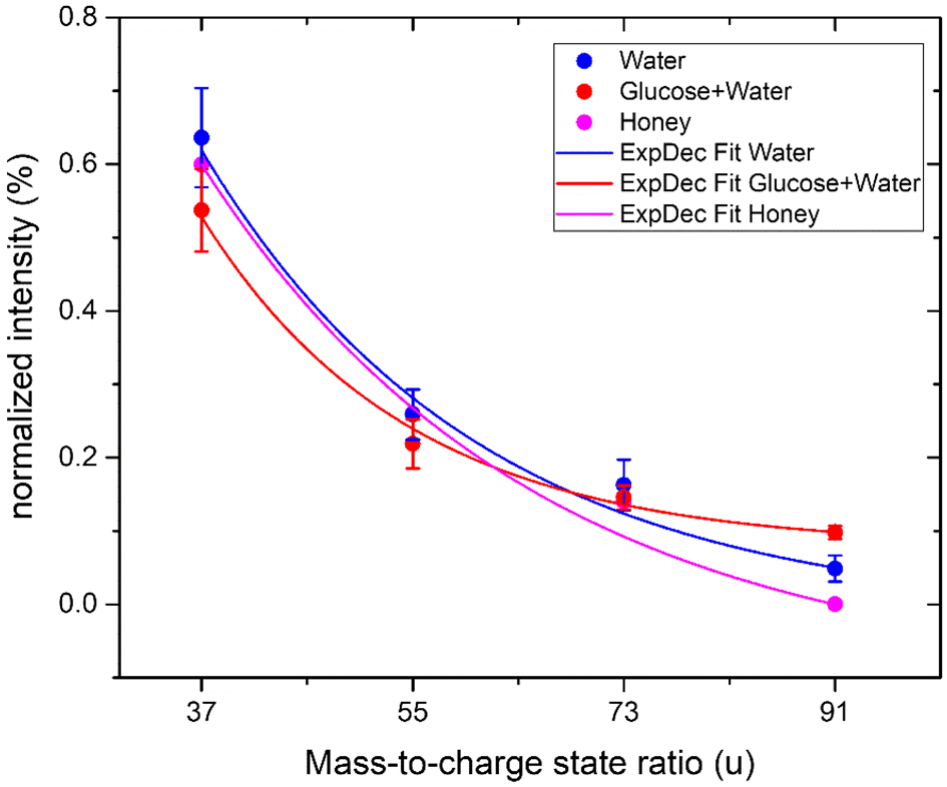
Relative peak intensities of the molecule (H_2_O)_*n*_H^+^ with *n* = 1–5 from a pure water measurement, glucose solution and honey are plotted against the respective mass-to-charge state ratio.

A direct comparison of the mass spectrum of honey to the previously measured pure water, saturated glucose solution and bulk solid glucose is presented in Fig. 2. All shown measurements have been performed under comparable laser conditions and evaporation rates. In water (blue) and honey (purple), we observe the same protonated water molecules (footprint signals) at m/q = 19, 37, 55 u, … that follow the molecular formula (H_2_O)_*n*_H^+^ with *n* = 1, 2, 3, … The observation of the same pronounced tailing (Fig. 3), as has been reported in earlier works for the footprint peaks^43,44^, confirms the peak assignment. However, the larger protonated clusters of this series at 73 and 91 u (*n* = 4, 5), which exist for pure water, are not observed in the honey spectra. This may be due to the lower water content of honey and the consequently higher evaporation field. Nevertheless, the honey spectra reveal peaks in the higher mass range up to m/q = 110 u, but these appear in multiple sets with a regular inter-peak distance of m/q = 1 u.

On the other hand, comparing the mass spectra of honey with those of a saturated glucose solution (red) significant differences become apparent, especially at higher mass-to-charge state ratios above 100 u. For the glucose solution, we had earlier identified a regular series of peaks as pyranose rings which had split off some OH + H fragments (see structure pictures in the top row). Interestingly, these peaks do not appear for honey, similar to the measurement of bulk sugar. Presumably, in both bulk solid glucose and honey, the evaporation field is already sufficient to split the pyranose rings of the glucose molecule or higher chain sugars. On the lower mass side, the signals of molecular origin are quite similar. An exception is the peak satellites in the range of m/q = 56–64, 66–76 and 82–90 u, which are more pronounced in the honey spectrum.

Comparing the mass spectrum of honey with bulk glucose (green), significant similarities can be observed. Consistently, no signals of molecules above m/q = 110 u are detected. Molecular fragments still dominate the mass spectrum but small signals of single atoms, C^+^ (12 u) and H^+^ (1 and 2 u) are also present. The peaks with the highest intensity for glucose bulk and honey are observed at m/q = 15 u corresponding to CH_3_^+^, at m/q = 29 u attributed to C_2_H_5_^+^ or COH^+^, at m/q = 44 u allowing multiple identification options (C_3_H_8_^+^, C_2_OH_4_^+^ or COO^+^). Only for honey, peaks at m/q = 58 u corresponding to C_4_H_10_^+^, C_3_OH_6_^+^ or C_2_O_2_H_2_^+^ can be observed. Other differences are naturally understood since bulk glucose does not contain water and therefore, the protonated water clusters (H_2_O)_*n*_H^+^ appear only for the solution. Also, the ratio of intensities between the peaks of H_2_O^+^ and H_3_O^+^ is indicative. Higher intensity at H_3_O^+^ is characteristic for water, which is not the case for the glucose bulk.

In summary, by comparing all four measurements with each other, it is obvious that besides the chemically necessary differences, the fragmentation behavior varies systematically from water to bulk glucose. Water and aqueous solutions tend to show large fragments and practically no atomic signals, while bulk glucose shows smaller fragments even some atomic species. In this respect, honey behaves closer to bulk glucose than to the aqueous solution, which can be attributed to the very high sugar fraction. This is macroscopically reflected by a high viscosity closer to a solid than to a thin fluid. Without a detailed calculation of the bonding strengths under high field conditions, the reason for the different fragmentation behaviour must remain partly speculative. In general, one can expect the different field strengths to be responsible, which are established during the measurement to reach the pre-set detection rate.

Besides, this may be also affected by different laser absorption and/or thermal conductivity of the studied materials. The thermal conductivity of honey is dominated by the carbohydrates, which have a value of 0.392 ± 0.62% W/m K^55^ and is slightly lower than that of glucose solutions or glucose crystals which both are in the range of 0.5 ± 0.1% W/m K^56,57^. The values reported for ice can be 5–10 times higher depending on the crystal structure, but the lowest value for amorphous ice at 0.6 W/m K^58^ is close to the solutions. Thus, variation in thermal conductivity can hardly explain the observed differences. No information is available on different light absorption under high field conditions. In future, different wavelengths need to be investigated in studying this phenomenon. So, it is likely that the established evaporation field is predominantly controlled by the difference in chemical bonding^59^. The bonding in natural honey is already closer to bulk glucose than to glucose solution.

In general, the mechanism of field evaporation for organic species is more complex than for metals, leading to molecular fragments of C_*x*_H_*y*_/C_*x*_O_*y*_H_*z*_ groups instead of single atoms. In our various APT experiments with soft matter and liquids and related DFT-based calculations^20,21,43,44,53^, we gathered increasing evidence that the field strength during the continuous field desorption is the decisive factor which in turn is mostly affected by the bonding strength of the matrix materials. For example, alkane chains strongly bonded by a thiol group to a metallic substrate evaporate in rather small hydrocarbons with one or two carbon atoms. The size of the evaporated fragments becomes larger with increasing laser power which reduces the field strength^20^. On the other hand, strong covalent intramolecular bonds, but a weak intermolecular van-der-Waals interaction leads to evaporation at low field strength and the detection of complete molecular units. This is demonstrated in the field evaporation of full *n*-tetradecane chains out of the van-der-Waals bonded frozen liquids^21^. Such evaporation of the whole molecule allows easy identification of the molecules as well as their localization in the reconstructed volume^43^, while detection of smaller fragments would theoretically improve the spatial resolution of the reconstruction, nevertheless, the overlap of peaks would be concentrated in a smaller number of mass signals. This would increase the uncertainty in the identification of individual peaks and thus reduce the accuracy of the measurement. So, weakly bonded single sugar molecules solved in water allow detection of the full sugar molecules, while the stronger inter-molecule bonds of crystalline sugar result in hydrocarbon fragments.

### Determination of compositional information

Information on the local stoichiometry of materials is important to understand the microstructure or to quantify transport coefficients and segregation factors. In the case of natural honey, the average composition is not exactly known. The possible quantitative evaluation and limitations will be discussed below.

The sugar content of honey consists of two different monosaccharides, glucose, and fructose, which have the same chemical formula C_6_O_6_H_12_, and of disaccharides (sucrose and maltose). In addition, other polypeptides, pollen, and minerals are contained in a small fraction. The complex composition makes the quantitative analysis challenging. So, the concept developed in previous work^43^ for peak identification of sugar solutions based on the expected ratio between water and glucose molecules, is attempted to be applied to the honey measurement. This concept is limited and can only be used to calculate the water content in the considered data set. The absence of intact sugar molecules in the spectrum prevents information about the distribution and composition of the different types of sugars, besides the fact that glucose and fructose cannot be distinguished in terms of their stoichiometry.

Following the concept of peak interpretation and convolution developed for the glucose solution^43^, three different interpretations of the observed mass peaks (see Table S3 in the supplementary information) are tested and the consequences on the overall stoichiometry on oxygen, are evaluated.

In the first approach, all signals detected in the honey measurement are interpreted as simple carbohydrates C_*x*_H_*z*_, except the peaks at m/q = 16, 17, 18 and 19 u as H_*x*_O^+^ with *x* = 0–3. This results in an extreme oxygen deficit of about 82.32%, which would be required to compensate the necessary oxygen atoms expected in the glucose rings (see Table 1). This amount cannot be simply explained by the typical loss of neutral oxygen during the APT measurement^54^.

**Table 1:** Calculated oxygen deficit and excess by different peak interpretations.

Interpretation	Oxygen deficit (%)	Oxygen excess (%)	Oxygen excess (wt%)
(C_*x*_H_*y*_)	82.32	–	–
(C_*x*_H_*y*_) + water peaks	50.67	–	–
(C_*x*_O_*y*_H_*z*_) + water peaks	–	14.84	36.5
(C_*x*_O_*y*_H_*z*_) + re-interpretation of 28–31 u + water peaks		7.36	18.7

In the second approach, we re-interpreted the peaks at 37, 55 and 73 u as the footprint molecules (H_2_O)_*n*_H^+^ with *n* = 1–3 found in previous experiments from the pure water measurement. With this, the apparent oxygen deficit is already reduced by a factor of 1.58 to compensate all oxygen atoms in the glucose molecules (Table 1).

In the third approach, part of the peaks is interpreted by combinations of C_*x*_O_*y*_H_*z*_, not anymore as C_*x*_H_*y*_ combinations, while keeping the footprint molecules as described in^44^. In this case, all oxygen atoms in all glucose molecules can be compensated and a slight excess of oxygen was estimated to be 14.84% (see Table 1). Corresponding to an H_2_O/glucose ratio of 36.5 wt.% which can be directly attributed to the water content.

With this, we can determine the water content of this measurement which is slightly higher than the values expected from literature (7.5–22.0 wt%) in Table S1 (see Supplementary Material).

However, this slight overestimation of the water content could be due to the difficult and not always ambiguous peak identification of the sugar fragments as either C_*x*_H_*y*_ or C_*x*_O_*y*_H_*z*_. The re-interpretation of the peaks at 28–31 u completely to C_2_H_4_^+^–C_2_H_7_^+^ instead of COH_*x*_ combinations results in 18.7 wt% of water which would be in the range of literature values. Due to the peak overlap of C_*x*_H_*y*_ and C_*x*_O_*y*_H_*z*_ molecules, the water content can be estimated in the range of 18.7–36.5 wt% depending on the proportion of these molecules to the peaks.

Still, one must not overlook the remaining sources of error in the determination of the actual water content. Numerous overlaps of sugar and water peaks occur in the mass spectra of honey (compare in Fig. 2 the mass spectra of pure water and glucose bulk with that of honey), which can lead to incorrect interpretation of the peaks. Currently, it is still impossible to resolve all overlaps, especially of single and double-charged ions at the same mass-to-charge state ratio, for example, C_3_^2+^ and H_2_O^+^ at the position at 18 u. There are efforts are underway though, to develop a kinetic energy-sensitive detector which could overcome such problems in the future^60^. But there are also some cases of overlap between different species, which have the same charge state. All these factors result in a plausible range rather than an accurate absolute value of the water content.

### Microstructural analysis

The outstanding feature of APT is a tomographic reconstruction of the measured volume with near-atomic resolution. With predominant evaporation of larger molecular species, the resolution is necessarily restricted by the size of the individual fragments. The volume is usually reconstructed by a geometrical algorithm from the recorded positions of detected ions. In the ideal case of pure metal, the reconstruction works quite well with the known algorithms^61,62^. Even atomic lattice resolution can be achieved. However, for the complex evaporation of organic materials, the current reconstruction algorithms may show limitations since the evaporation fields of the components differ vastly. To estimate the quality of obtained reconstructions, investigation of a heterogeneous microstructure is of advantage.

**Figure 4:**
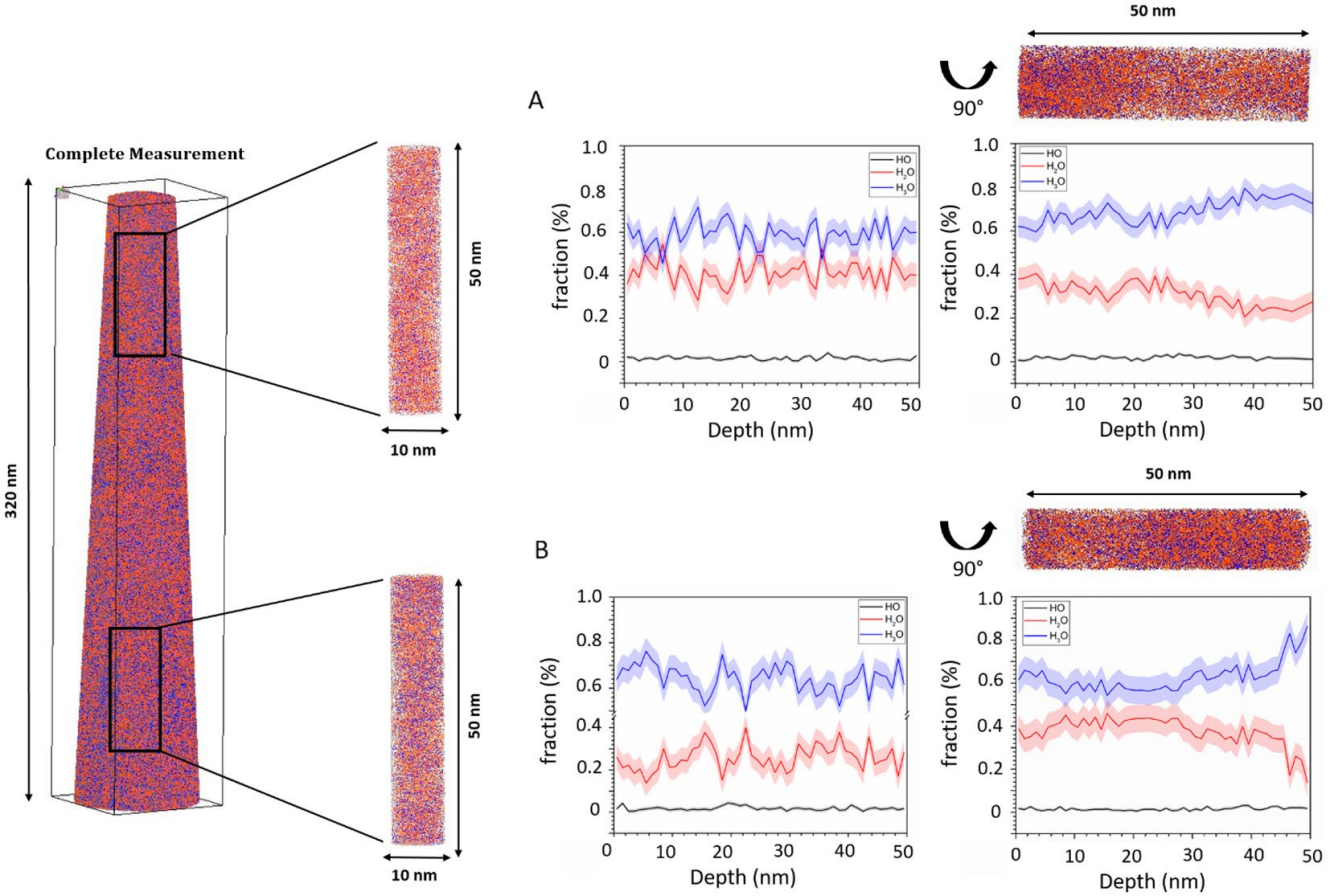
Representative 3D reconstruction of a measured specimen with a total length of 320 nm. A cylinder with a dimension of 10 × 10 × 50 nm was selected vertical and horizontal to the tip from the total volume to analyse the composition profile of the H_*x*_O molecules with *x* = 1–3.

To investigate the distribution of fragments stemming from different sugar types or water, the evaporated volume of honey was reconstructed from the detector data (see “Experimental section/methods” section for more details). The distribution of the individual signals of HO^+^, H_2_O^+^ and H_3_O^+^ (17, 18, and 19 u) molecules are probed by a cylinder with a dimension of 10 × 10 × 50 nm along the tip axis and perpendicular to the axis (at the same position rotated by 90°) at the beginning (Fig. 4A) of the measurement and the end (Fig. 4B). While the relative ratio of the water signals is almost constant in the axis direction, a significant change in the relative abundance of H_3_O^+^ to H_2_O^+^ is noticed perpendicular to the tip axis. The direction of laser incidence correlates with an increase in the H_2_O^+^ signal. Therefore, this phenomenon can be explained by the local absorption of the UV laser light and the consequent heterogeneous pulse temperature. In large band gap materials, it is known that there is a heat transfer from the laser incidence side to the shadow side^63^. On the other hand, it is well known that the tip curvature modifies in response, thus, a thermally increased probability of evaporation is compensated by a decrease in curvature and consequently a decrease in field strength^64,65^. This is usually reflected in a higher fraction of singly charged ions^64^. Therefore, a large fraction of singly charged ions indicates a locally increased temperature due to the laser impact. However, here it seems it also favours the evaporation of H_2_O^+^ molecules. Based on the desorption maps for the complete measurement as well as for selected molecules presented in Fig. S4, areas of higher intensity and line features related to the laser incidence direction can be observed, as reported in previous work on pure water and saturated glucose solution^43,44^.

**Figure 5:**
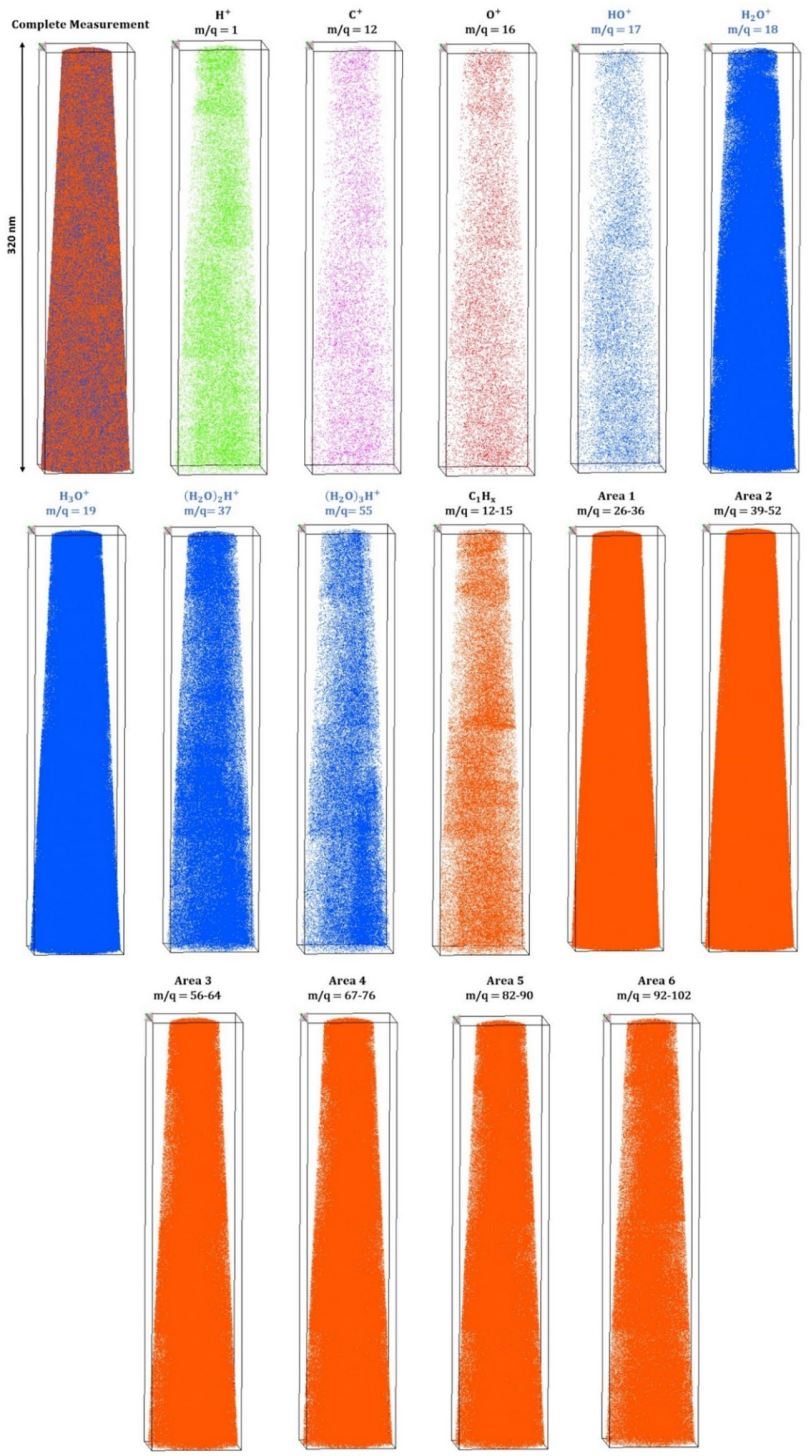
Individual maps of the reconstructed volume for different elements and molecules are shown.

Considering the distribution of the measured individual elements (see Fig. 5), these elements H, C and O seem to be homogeneously distributed in the volume. But the fraction of atomic H, C and O in the total measurement is only 1.06 at.%, which reveals that the splitting of the molecules into their elements is very rare. However, the water footprint molecules (H_2_O)_*n*_H^+^ with *n* = 0–3 show a certain localization, especially with higher masses (*n* = 3), which is also represented in the desorption maps (Fig. S4 in the supplementary material). The sugar fragments were subdivided into six groups to visualize them (Fig. 5).

**Figure 6:**
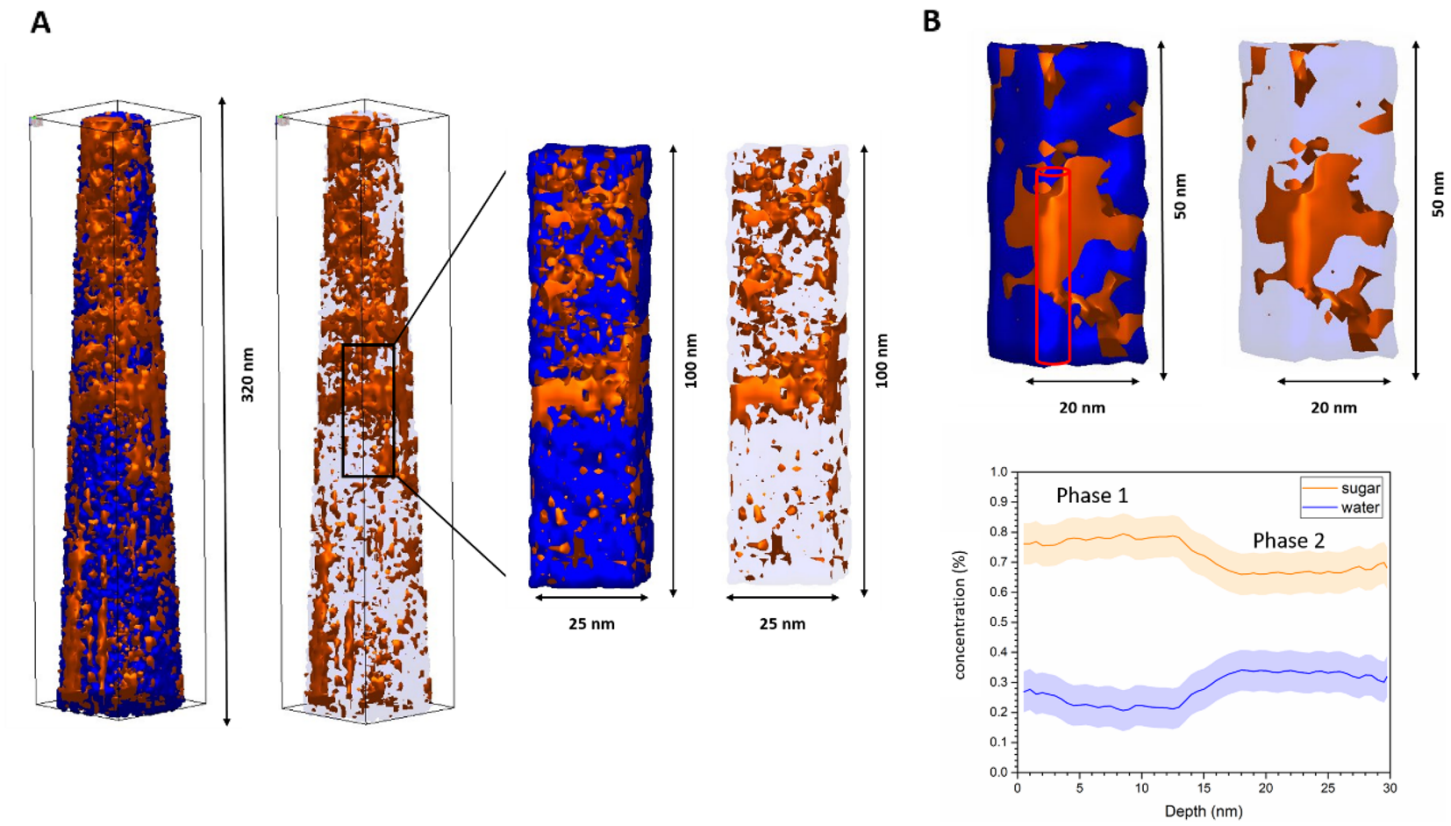
Isocomposition surfaces of water are shown in blue, and all fragments of sugar molecules or organic origin are shown in orange to visualize the agglomeration or the domains of nano-crystallinity (**A**). In (**B**), a composition profile (indicated by a red cylinder) along a sugar-rich region and the interface to a water-rich region was investigated. The sugar content in the orange region is significantly higher than in the blue region. Error bars were calculated with the standard deviation.

Nevertheless, the peaks HO^+^, H_2_O^+^, H_3_O^+^ and the protonated water clusters at m/q = 37 and 55 u were used to calculating the local water content, while all other peaks were assumed to stem from sugar components. In this way indeed, local regions with enrichment of water can be visualized. Because honey is a supercritical solution of different sugar molecules, with a total water content of about 10–20 wt%, it can be assumed that the sugar molecules cannot homogeneously dissolve in water, but nanocrystals and precipitates might have formed. In Fig. 6, regions with sugar content above an isocompositon threshold of 75% (water-related peaks/sugar fragments C_*x*_O_*y*_H_*z*_) are visualized by an orange isocomposition surface, whereas the water-rich regions of a least 25% sugar are represented by a second isocomposition surface in blue. Besides mere equilibrium decomposition, also kinetic processes might appear e.g., segregation at the crystallization front of sugar during the freezing process. However, none of these processes could be observed in the measurement of quench-frozen saturated glucose solution. Many areas with an accumulation of sugar fragments can be found, which indicates agglomeration and corresponds to a nano-crystallinity of honey, which is in principle known^50,51^ and now can be demonstrated by APT.

Due to the different solubility limits of glucose (lower solubility) and fructose (higher solubility) in water, glucose precipitates and crystallizes more rapidly. This results in two phases, a crystalline phase, and a liquid phase, with different compositions^50,51^. The liquid phase has therefore a higher content of fructose and the crystalline phase has a higher content of glucose due to the crystallization. Since glucose and fructose have the same molecular formula, it is impossible to distinguish between fructose and glucose, which makes the investigation of the two phases concerning the composition of different sugars unfeasible. The difference between the glucose concentration in the crystalline phase and the liquid phase is about 30% depending on the type of honey^50,51^. Due to the crystallization of glucose, the water content in the remaining liquid phase increases. This difference in the composition of the two phases and the unambiguous identification of the fingerprint water molecules allows us to visualize and identify different phases with the help of two different isocomposition surfaces (Fig. 6A).

In order the analyse the composition of the different phases, as well as the presence of a nanocrystalline precipitates of glucose, a representative box with a length of 50 nm and a square width of 20 nm was selected from the entire volume. A composition profile along the red cylinder marked in Fig. 6B was analysed and qualitatively proves the presence of nanoprecipitations.

The sugar concentration of the solid phase could be qualified above 77.45 ± 1.24% of C_*x*_O_*y*_H fragments (23.15 ± 2.04% of water-related peaks), whereas the remaining liquid phase has only a sugar content of about 67.03 ± 1.01% C_*x*_O_*y*_H_*z*_ molecules (32.97 ± 1.08% water-related peaks) and can therefore clearly be used to identify and distinguish both phases.

**Figure 7:**
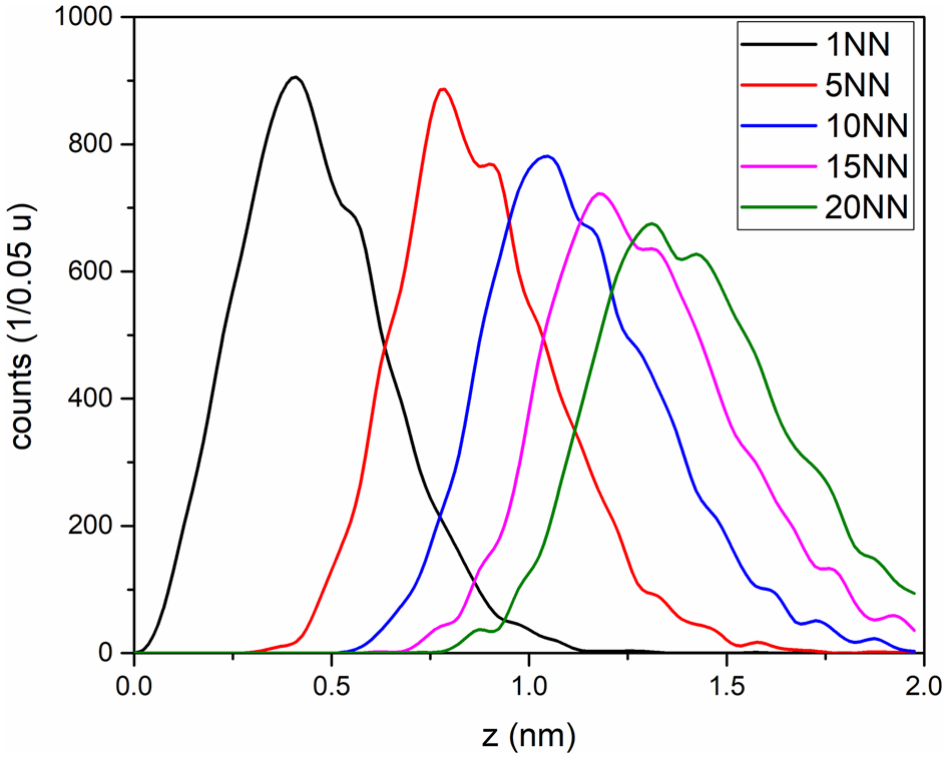
Radius distribution to include sugar molecules NN around a sugar molecule to identify the different distances within both phases.

To prove that two different phases are formed, a nearest-neighbour (NN) analysis was performed. By determining the distance of the next neighbours of increasing order between all sugar fragments, the distribution of sugar molecules in solution, and in the box of interest can be statistically analysed. Two maxima are visible (Fig. 7), which are significant as they appear for all neighbour orders (1, 5, 10, 15 and 20 NN) and indicate that the sugar molecules have a preferential separation. The sugar molecules have a shorter distance to each other in the solid phase as in the liquid phase, which proves the evidence of the two phases.

**Figure 8:**
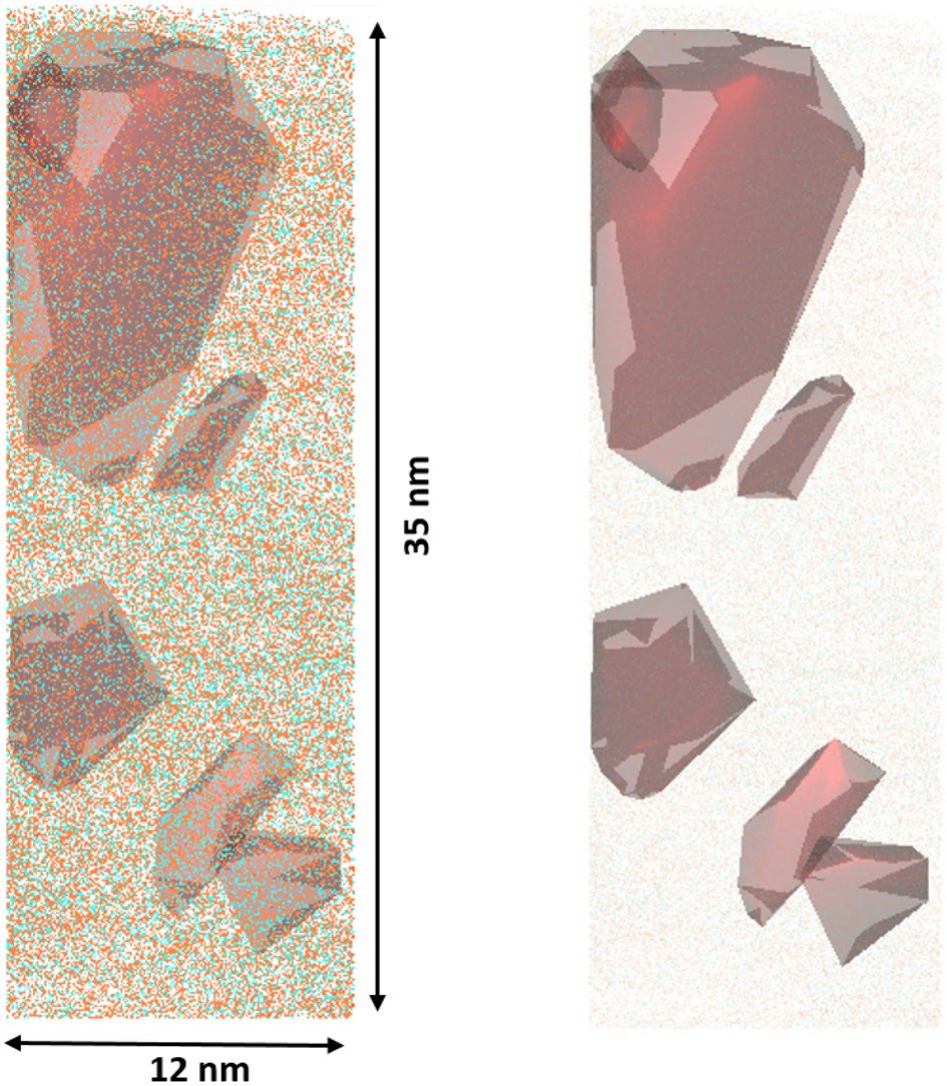
The sugar cluster is visualized by red areas into a defined box with a volume of 12 × 12 × 35 nm. In total six clusters with varied sizes and shapes can be identified in this volume.

Based on the NN curves in Fig. 7, a cluster search algorithm can be used to visualize the clusters of sugar-containing components within this box of interest (Fig. 8). The average concentration of sugar molecules (C_*x*_O_*y*_H_*z*_) in the clusters is 72.02 ± 3.54% and the water content (water-related peaks) is correspondingly 27.98 ± 3.54%, which is already in the range shown in the composition profile and proves the evidence of two phases separation with different sugar ratios. The analysed z-direction of the crystalline phase could be determined in the box of interest to be 4.36–15.78 nm with a respective volume of the clusters between 12 and 567 nm^3^, but this large range indicates that the size and the shape of the crystalline phases vary widely. In addition, nothing is known about the shape and size of the precipitation in the literature.

Besides many difficulties and progress which is still required, the attempt to freeze and measure a complex organic liquid show that it is possible to identify and localize nanostructures in liquids and paves the way for embedding and analyzing nanostructures or other features of interest in aqueous liquids.

In future, by embedding features e.g., nanoparticles, virus envelope, biomolecules … with known structures from correlative methods such as cryo-TEM into an aqueous environment, the reconstruction of the obtained APT data from aqueous samples should be calibrated and its accuracy in terms of local and chemical resolution should be investigated. If necessary, the existing reconstruction algorithm should be adapted and modified.

## Conclusion

We have demonstrated in this work, that a structural analysis of frozen soft matter samples in the nanoscale range is achievable with APT. Honey was analyzed as a complex organic high viscous liquid, and the mass spectra were compared to pure water, saturated glucose solution and glucose bulk to close the chain of evidence. Honey shows more similarities in mass spectra to glucose bulk than to the aqueous saturated solution, which is due to the very high sugar content, reflected macroscopically in high viscosity and reminiscent closer to a solid than a liquid. The water content was determined using the footprint molecules of water found in previous measurements and is about 18.7–36.5 wt%, which is in the range of the usual moisture content in honey reported in several works of literature. Using isocomposition surfaces and a composition profile, the two different phases in honey described in the literature could be identified and localized based on their different sugar concentrations. Both phases could be proven by NN analysis and a cluster search algorithm. The average concentration of sugar components in the crystalline phase is determined to be 77.45 ± 1.24% (C_*x*_O_*y*_H_*z*_) and the water content is correspondingly 23.15 ± water-related peaks. The size of the clusters in the box of interest was determined to be 4.36–15.78 nm with a respective volume of the clusters between 12 and 567 nm^3^. This demonstrated for the first time that organic nanostructures can be analyzed and localized in frozen solutions. Furthermore, nanostructures can be localized in frozen aqueous solutions, enabling the establishment and further development in the measurement of soft matter and especially frozen aqueous specimens in atom probe tomography.

## Experimental section/methods

### Materials and freezing process

**Figure 9:**
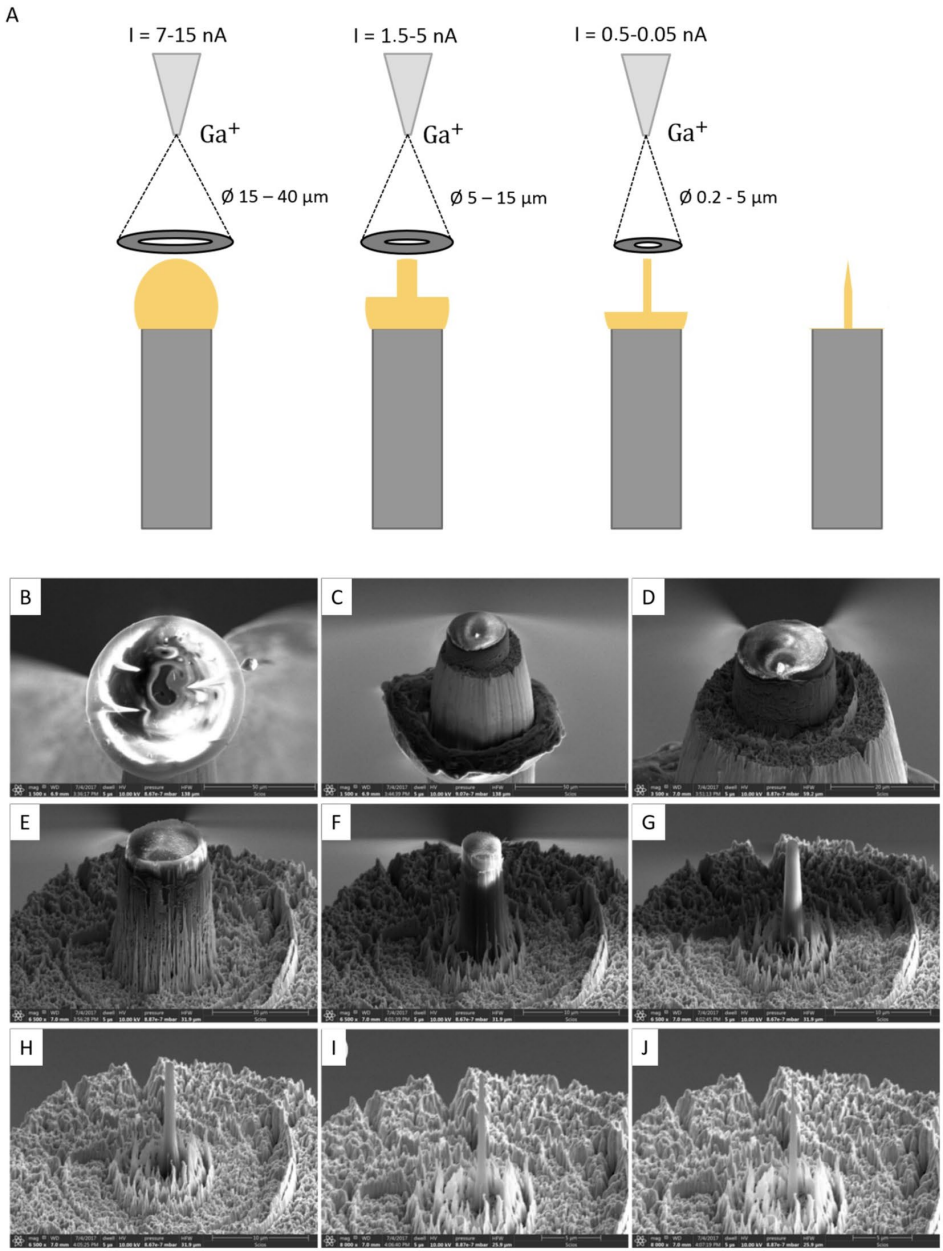
FIB Preparation—above in (**A**) the annular milling process with a decreasing inner diameter and respective current is schematically shown. (**B**) Frozen honey droplet on top of the tungsten post. (**A**–**I**) show the milling process with an annular pattern and decreasing inner diameter until a final tip with a radius < 100–200 nm (**J**) is obtained.

As sample material, natural honey from the company Langnese was used. Tungsten wires were used as substrate material and their rough surfaces were produced by applying a tensile force under cryogenic conditions^43,44,66^. It’s well known that honey has a high viscosity and, therefore, sticks to different surfaces. In the case of honey, the rough tungsten wire, was initially dipped into the honey and afterwards quenched to cryogenic temperature into a liquid N_2_ bath. The obtained honey droplet with a diameter of 50 µm can be seen in Fig. 9B. This method cannot be used for low viscous liquids; therefore, a dedicated method was developed as reported in^43,44,66^. After that, the sample holder was transferred as fast as possible into the body of the cooled transfer shuttle VCT500 from Leica (T = − 184 °C). This system allowed to transfer of samples at cryo-temperatures (T = − 184 °C) and under a low-pressure atmosphere (10^–4^ mbar) between the machines without contaminations from the environment.

### Cryo FIB preparation

To prepare frozen droplets in the FIB (FEI Scios) into nano-shaped tips (r < 100 nm), which are necessary for the APT method, the FIB has been equipped with a custom made cryo-stage, which is cooled down to a temperature of − 150 °C by copper bands connected to an N_2_ Dewar which is attached to the FIB. SEM imaging was typically performed with low energy (5 kV 25 pA) to prevent melting of the sample by electron bombardment. A circular ring pattern was used for azimuthal milling. Initial milling steps were performed at an acceleration voltage of 30 kV and an ion beam current of 15 nA until the tungsten substrate became visible. After reaching a radius of 30 µm, the beam current is gradually reduced with decreasing diameter, down to an inner ring diameter of 300 nm and a beam current of 0.1 pA. The shaping process continues until a very sharp tip with a radius < 100 nm (Fig. 9) is obtained^67^. The finished tip is then transferred back into the shuttle and is attached to the APT. A tip length of several tens of µm is required.

### APT measurements

The presented results were obtained by using a custom-made atom probe^68^ operating at a laser wavelength of 355 nm. The pulse length accounts for 250 fs and the spot size diameter to 50 microns. The system is equipped with a 120 mm diameter delay line detector with an open area ratio (OAR) of 50%. The system was additionally equipped with a custom-made cryo-transfer port to accept a standard VCT500 from Leica for the transfer of cryogenic samples.

### Reconstruction

The obtained datasets were analyzed using the Scito^69^ software package. The volume was reconstructed using a geometrical reconstruction algorithm^70^ based on the original point projection method by Bas et al.^61^. SEM images were used to determine the initial tip radius and taper angle. The tip radius and taper angle were used to optimize the calculated evaporation field curve to be in the range of the field strength of water which is about 8–12 V/nm^53^. All necessary parameters, such as field and image compression factors were determined in earlier measurements.

## Supplementary Information

Supplementary Information.

## Data Availability

All data needed to evaluate the conclusions in the paper are included in the paper and/or supplementary materials. Additional data for this work can be requested from the contributed author T.M.S.
