# Supplementary material for: Nanoscale analysis of frozen honey by atom probe tomography
Source: Sci Rep. 2022 Oct 22;12:17786. doi: 10.1038/s41598-022-22717-9 (PMC9587987; doi:10.1038/s41598-022-22717-9)
Supplement: Supplementary file 1 — Supplementary Information. [file 41598_2022_22717_MOESM1_ESM.docx]

Supplementary Materials for

**Nanoscale Analysis of Frozen Honey by Atom Probe Tomography**

Tim.M. Schwarz^*^, Jonas Ott, Helena Solodenko, Guido Schmitz, Patrick Stender

*Corresponding author. Email: tim.schwarz@mp.imw.uni-stuttgart.de

**This PDF file includes:**

Tables S1 to S3

Fig. S1 to S5

Table S 1: Comparison of different contents of honey (Apis mellifera L.) regarding to different countries *(48)*.

| Continents/countries | Sugars (g 100 g^-1^)  Fructose Glucose Sucrose | | | Moisture (g 100 g^-1^) | References |
| --- | --- | --- | --- | --- | --- |
| European/Spain | 37.75-41-40 | 28.80-37.30 | 0.15-1.43 | 15.40-17.38 | Manzanares et al. (2014) |
| European/Spain,Romania and Czech Republic | 39.30-49.20 | 26.80-38.30 | 0.60-2.20 | 15.30-17.50 | Juan-Borras et al. (2014) |
| European/Greece | - | - | - | 10.47-20.47 | Karabagias et al. (2014) |
| African/Morocco | 39.44-42.42 | 29.25-33.08 | 0.47-1.86 | 14.64-18.59 | Chakir et al. (2011) |
| African/Tunisia | 35.78-37.84 | 31.07-36.58 | n.d. -4.60 | 17.27-19.80 | Boussaid et al (2018) |
| African-European/ Turkey | 29.80-44.49 | 25.93-35.98 | 2.85-8.44 | 7.99-17.40 | Tornuk et al. (2013) |
| Asian/Indian | 43.30-65.50 | - | 0.40-8.80 | 17.20-21.60 | Sayena et al. (2010) |
| American/Argentina | 67.70-73.5 | - | 0.40-5.60 | 14.10-18.80 | Isal et al. (2011) |
| American/Uruguay | . | . | . | 16.60-18.61 | Corbella and Cozzolino (2006) |
| American/Brazil | 33.30-38.60 | 21.00-26.35 | 0.12-0.50 | 17.10-20.50 | Moreira et al. (2007) |
| American/USA | 30.9-44.3 | 22.9-40.7 | 0.2-7.6 | 12.2-22.9 | Ball (2007) |

Table S 2: Possible combinations of different C*_x_*H*_y_*, C*_x_*O*_y_*H*_z_* and H*_x_*O*_y_* molecules in honey considering only the single charged state.

| Peak identification |  |  |  |  |  |
| --- | --- | --- | --- | --- | --- |
| m/q (u) | **molecules** |  |  |  |  |
| 1 | H |  |  |  |  |
| 12 | C |  |  |  |  |
| 14 | CH2 |  |  |  |  |
| 15 | CH3 |  |  |  |  |
| 16 | O |  |  |  |  |
| 17 | OH |  |  |  |  |
| 18 | OH2 |  |  |  |  |
| 19 | OH3 |  |  |  |  |
| 27 | C2H3 |  |  |  |  |
| 28 | C2H4 | CO |  |  |  |
| 29 | C2H5 | COH |  |  |  |
| 30 | C2H6 | COH2 |  |  |  |
| 31 | C2H7 | COH3 |  |  |  |
| 32 | O2 |  |  |  |  |
| 33 | O2H |  |  |  |  |
| 34 | (OH)2 |  |  |  |  |
| 35 | (OH)(OH2) |  |  |  |  |
| 36 | (OH2)2 | C3 |  |  |  |
| 37 | (H2O)(H3O) | C3H |  |  |  |
| 38 |  | C3H2 |  |  |  |
| 39 |  | C3H3 |  |  |  |
| 40 |  | C3H4 | C2O |  |  |
| 41 |  | C3H5 | C2OH |  |  |
| 42 |  | C3H6 | C2OH2 |  |  |
| 43 |  | C3H7 | C2OH3 |  |  |
| 44 | CO2 | C3H8 | C2OH4 |  |  |
| 45 | CO2H | C3H9 | C2OH5 |  |  |
| 46 | CO2H2 |  | C2OH6 |  |  |
| 47 | CO2H3 |  | C2OH7 |  |  |
| 48 | CO2H4 | C4 |  |  |  |
| 49 | CO2H5 | C4H |  |  |  |
| 50 |  | C4H2 |  |  |  |
| 51 |  | C4H3 |  |  |  |
| 52 |  | C4H4 | C3O |  |  |
| 53 |  | C4H5 | C3OH |  |  |
| 54 |  | C4H6 | C3OH2 |  |  |
| 55 | (H2O)2(H3O) | C4H7 | C3OH3 |  |  |
| 56 |  | C4H8 | C3OH4 | C2O2 |  |
| 57 |  | C4H9 | C3OH5 | C2O2H |  |
| 58 |  | C4H10 | C3OH6 | C2O2H2 |  |
| 59 |  | C4H11 | C3OH7 | C2O2H3 |  |
| 60 | C5 |  | C3OH8 | C2O2H4 |  |
| 61 | C5H |  | C3OH9 | C2O2H5 |  |
| 62 | C5H2 |  |  | C2O2H6 |  |
| 63 | C5H3 |  |  | C2O2H7 |  |
| 64 | C5H4 |  | C4O |  |  |
| 65 | C5H5 |  | C4OH |  |  |
| 66 | C5H6 |  | C4OH2 |  |  |
| 67 | C5H7 |  | C4OH3 |  |  |
| 68 | C5H8 |  | C4OH4 | C3O2 |  |
| 69 | C5H9 |  | C4OH5 | C3O2H |  |
| 70 | C5H10 |  | C4OH6 | C3O2H2 |  |
| 71 | C5H11 |  | C4OH7 | C3O2H3 |  |
| 72 | C5H12 | C6 | C4OH8 | C3O2H4 |  |
| 73 | C5H13 | C6H | C4OH9 | C3O2H5 |  |
| 74 |  | C6H3 | C4OH10 | C3O2H6 |  |
| 75 |  | C6H4 | C4OH11 | C3O2H7 |  |
| 76 |  | C6H5 | C5O | C3O2H8 |  |
| 77 |  | C6H6 | C5OH | C3O2H9 |  |
| 78 |  | C6H7 | C5OH2 |  |  |
| 79 |  | C6H8 | C5OH3 |  |  |
| 80 |  | C6H9 | C5OH4 |  | C4O2 |
| 81 |  | C6H10 | C5OH5 |  | C4O2H |
| 82 |  | C6H11 | C5OH6 |  | C4O2H2 |
| 83 |  | C6H12 | C5OH7 |  | C4O2H3 |
| 84 | C3O3 | C6H13 | C5OH8 |  | C4O2H4 |
| 85 | C3O3H | C6H14 | C5OH9 |  | C4O2H5 |
| 86 | C3O3H2 | C6H15 | C5OH10 |  | C4O2H6 |
| 87 | C3O3H3 |  | C5OH11 |  | C4O2H7 |
| 88 | C3O3H4 |  | C5OH12 | C6O | C4O2H8 |
| 89 | C3O3H5 |  | C5OH13 | C6OH | C4O2H9 |
| 90 | C3O3H6 |  |  | C6OH2 | C4O2H10 |
| 91 | C3O3H7 |  |  | C6OH3 | C4O2H11 |
| 92 | C3O3H8 |  |  | C6OH4 | C5O2 |
| 93 | C3O3H9 |  |  | C6OH5 | C5O2H |
| 94 |  |  |  | C6OH6 | C5O2H2 |
| 95 |  |  |  | C6OH7 | C5O2H3 |
| 96 |  |  | C4O3 | C6OH8 | C5O2H4 |
| 97 |  |  | C4O3H | C6OH9 | C5O2H5 |
| 98 |  |  | C4O3H2 | C6OH10 | C5O2H6 |
| 99 |  |  | C4O3H3 | C6OH11 | C5O2H7 |
| 100 |  |  | C4O3H4 | C6OH12 | C5O2H8 |
| 101 |  |  | C4O3H5 | C6OH13 | C5O2H10 |
| 102 |  |  | C4O3H6 | C6OH14 | C5O2H11 |
| 103 |  |  | C4O3H7 | C6OH15 | C5O2H12 |

Table S 3: For the first approach only C*_x_*H*_y_* molecules are considered. In the second approach C*_x_*H*_y_* molecules and common protonated water cluster with the formula (H_2_O)*_n_*H^+^ with *n* = 1 -5 were assumed. And in the third approach C*_x_*O*_y_*H*_z_* and protonated water were supposed.

| Approach 1 C*_x_*H*_y_* | | Approach 2 C*_x_*H*_y_* + Water Peaks | | Approach 3 C*_x_*O*_y_*H*_z_* + Water Peaks | |
| --- | --- | --- | --- | --- | --- |
| m/q (u) | **molecules** | **m/q (u)** | **molecules** | **m/q (u)** | **molecules** |
| 1 | H | **1** | H | **1** | H |
| 12 | C | **12** | C | **12** | C |
| 14 | CH2 | **14** | CH2 | **14** | CH2 |
| 15 | CH3 | **15** | CH3 | **15** | CH3 |
| 16 | O | **16** | O | **16** | O |
| 17 | OH | **17** | OH | **17** | OH |
| 18 | OH2 | **18** | OH2 | **18** | OH2 |
| 19 | OH3 | **19** | OH3 | **19** | OH3 |
| 27 | C2H3 | **27** | C2H3 | **27** | C2H3 |
| 28 | C2H4 | **28** | C2H4 | **28** | CO |
| 29 | C2H5 | **29** | C2H5 | **29** | COH |
| 30 | C2H6 | **30** | C2H6 | **30** | COH2 |
| 31 | C2H7 | **31** | C2H7 | **31** | COH3 |
| 32 | O2 | **32** | O2 | **32** | O2 |
| 33 | O2H | **33** | O2H | **33** | O2H |
| 34 | (OH)2 | **34** | (OH)2 | **34** | (OH)2 |
| 35 | (OH)(OH2) | **35** | (OH)(OH2) | **35** | (OH)(OH2) |
| 36 | C3 | **36** | (OH2)2 | **36** | (OH2)2 |
| 37 | C3H | **37** | (H2O)(H3O) | **37** | (H2O)(H3O) |
| 38 | C3H2 | **38** | C3H2 | **38** | C3H2 |
| 39 | C3H3 | **39** | C3H3 | **39** | C3H3 |
| 40 | C3H4 | **40** | C3H4 | **40** | C2O |
| 41 | C3H5 | **41** | C3H5 | **41** | C2OH |
| 42 | C3H6 | **42** | C3H6 | **42** | C2OH2 |
| 43 | C3H7 | **43** | C3H7 | **43** | C2OH3 |
| 44 | C3H8 | **44** | C3H8 | **44** | CO2 |
| 45 | C3H9 | **45** | C3H9 | **45** | CO2H |
| 46 | C2OH6 | **46** | C2OH6 | **46** | CO2H2 |
| 47 | C2OH7 | **47** | C2OH7 | **47** | CO2H3 |
| 48 | C4 | **48** | C4 | **48** | CO2H4 |
| 49 | C4H | **49** | C4H | **49** | CO2H5 |
| 50 | C4H2 | **50** | C4H2 | **50** | C4H2 |
| 51 | C4H3 | **51** | C4H3 | **51** | C4H3 |
| 52 | C4H4 | **52** | C4H4 | **52** | C3O |
| 53 | C4H5 | **53** | C4H5 | **53** | C3OH |
| 54 | C4H6 | **54** | C4H6 | **54** | C3OH2 |
| 55 | C4H7 | **55** | (H2O)2(H3O) | **55** | (H2O)2(H3O) |
| 56 | C4H8 | **56** | C4H8 | **56** | C2O2 |
| 57 | C4H9 | **57** | C4H9 | **57** | C2O2H |
| 58 | C4H10 | **58** | C4H10 | **58** | C2O2H2 |
| 59 | C4H11 | **59** | C4H11 | **59** | C2O2H3 |
| 60 | C5 | **60** | C5 | **60** | C2O2H4 |
| 61 | C5H | **61** | C5H | **61** | C2O2H5 |
| 62 | C5H2 | **62** | C5H2 | **62** | C2O2H6 |
| 63 | C5H3 | **63** | C5H3 | **63** | C2O2H7 |
| 64 | C5H4 | **64** | C5H4 | **64** | C4O |
| 65 | C5H5 | **65** | C5H5 | **65** | C4OH |
| 66 | C5H6 | **66** | C5H6 | **66** | C4OH2 |
| 67 | C5H7 | **67** | C5H7 | **67** | C4OH3 |
| 68 | C5H8 | **68** | C5H8 | **68** | C3O2 |
| 69 | C5H9 | **69** | C5H9 | **69** | C3O2H |
| 70 | C5H10 | **70** | C5H10 | **70** | C3O2H2 |
| 71 | C5H11 | **71** | C5H11 | **71** | C3O2H3 |
| 72 | C5H12 | **72** | C5H12 | **72** | C3O2H4 |
| 73 | C6H | **73** | C6H | **73** | C3O2H5 |
| 74 | C6H3 | **74** | C6H3 | **74** | C3O2H6 |
| 75 | C6H4 | **75** | C6H4 | **75** | C3O2H7 |
| 76 | C6H5 | **76** | C6H5 | **76** | C3O2H8 |
| 77 | C6H6 | **77** | C6H6 | **77** | C3O2H9 |
| 78 | C6H7 | **78** | C6H7 | **78** | C5OH2 |
| 79 | C6H8 | **79** | C6H8 | **79** | C5OH3 |
| 80 | C6H9 | **80** | C6H9 | **80** | C4O2 |
| 81 | C6H10 | **81** | C6H10 | **81** | C4O2H |
| 82 | C6H11 | **82** | C6H11 | **82** | C4O2H2 |
| 83 | C6H12 | **83** | C6H12 | **83** | C4O2H3 |
| 84 | C6H13 | **84** | C6H13 | **84** | C3O3 |
| 85 | C6H14 | **85** | C6H14 | **85** | C3O3H |
| 86 | C6H15 | **86** | C6H15 | **86** | C3O3H2 |
| 87 | C5OH11 | **87** | C5OH11 | **87** | C3O3H3 |
| 88 | C6O | **88** | C6O | **88** | C3O3H4 |
| 89 | C6OH | **89** | C6OH | **89** | C3O3H5 |
| 90 | C6OH2 | **90** | C6OH2 | **90** | C3O3H6 |
| 91 | C6OH3 | **91** | C6OH3 | **91** | C3O3H7 |
| 92 | C6OH4 | **92** | C6OH4 | **92** | C3O3H8 |
| 93 | C6OH5 | **93** | C6OH5 | **93** | C3O3H9 |
| 94 | C6OH6 | **94** | C6OH6 | **94** | C5O2H2 |
| 95 | C6OH7 | **95** | C6OH7 | **95** | C5O2H3 |
| 96 | C6OH8 | **96** | C6OH8 | **96** | C4O3 |
| 97 | C6OH9 | **97** | C6OH9 | **97** | C4O3H |
| 98 | C6OH10 | **98** | C6OH10 | **98** | C4O3H2 |
| 99 | C6OH11 | **99** | C6OH11 | **99** | C4O3H3 |
| 100 | C6OH12 | **100** | C6OH12 | **100** | C4O3H4 |
| 101 | C6OH13 | **101** | C6OH13 | **101** | C4O3H5 |
| 102 | C6OH14 | **102** | C6OH14 | **102** | C4O3H6 |
| 103 | C6OH15 | **103** | C6OH15 | **103** | C4O3H7 |





Figure S 1: Voltage curve of the APT measurement.


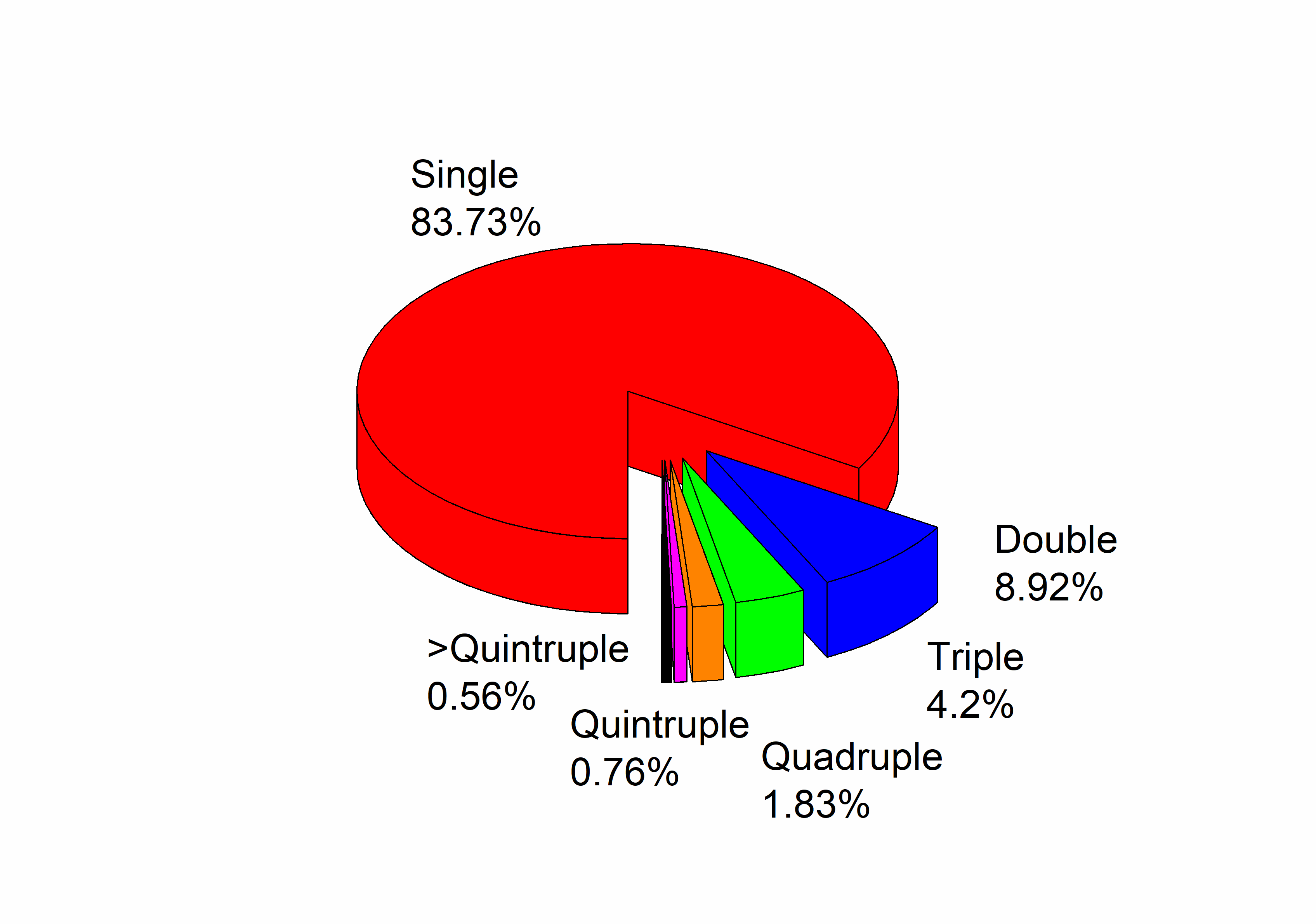


Figure S 2: Number of multi-hit events of the measurement in percentage.


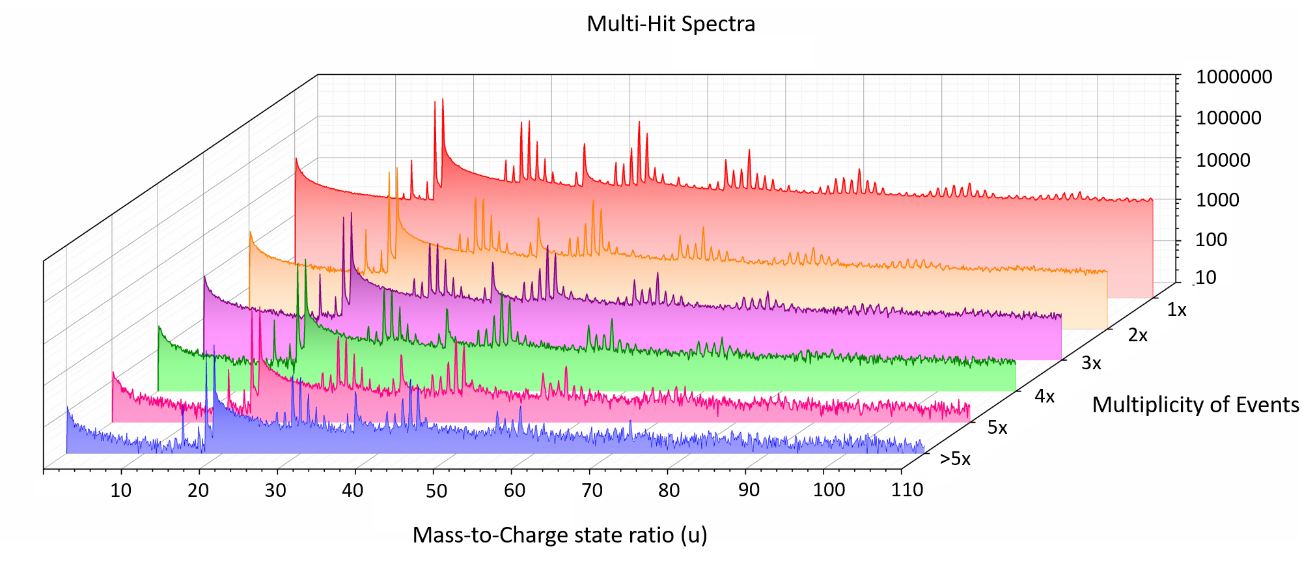


Figure S 3: Mass spectrum split into single, double, triple, quadruple, quintruple and >quintruple events from the mass-to-charge state ratio of 0-110 u.


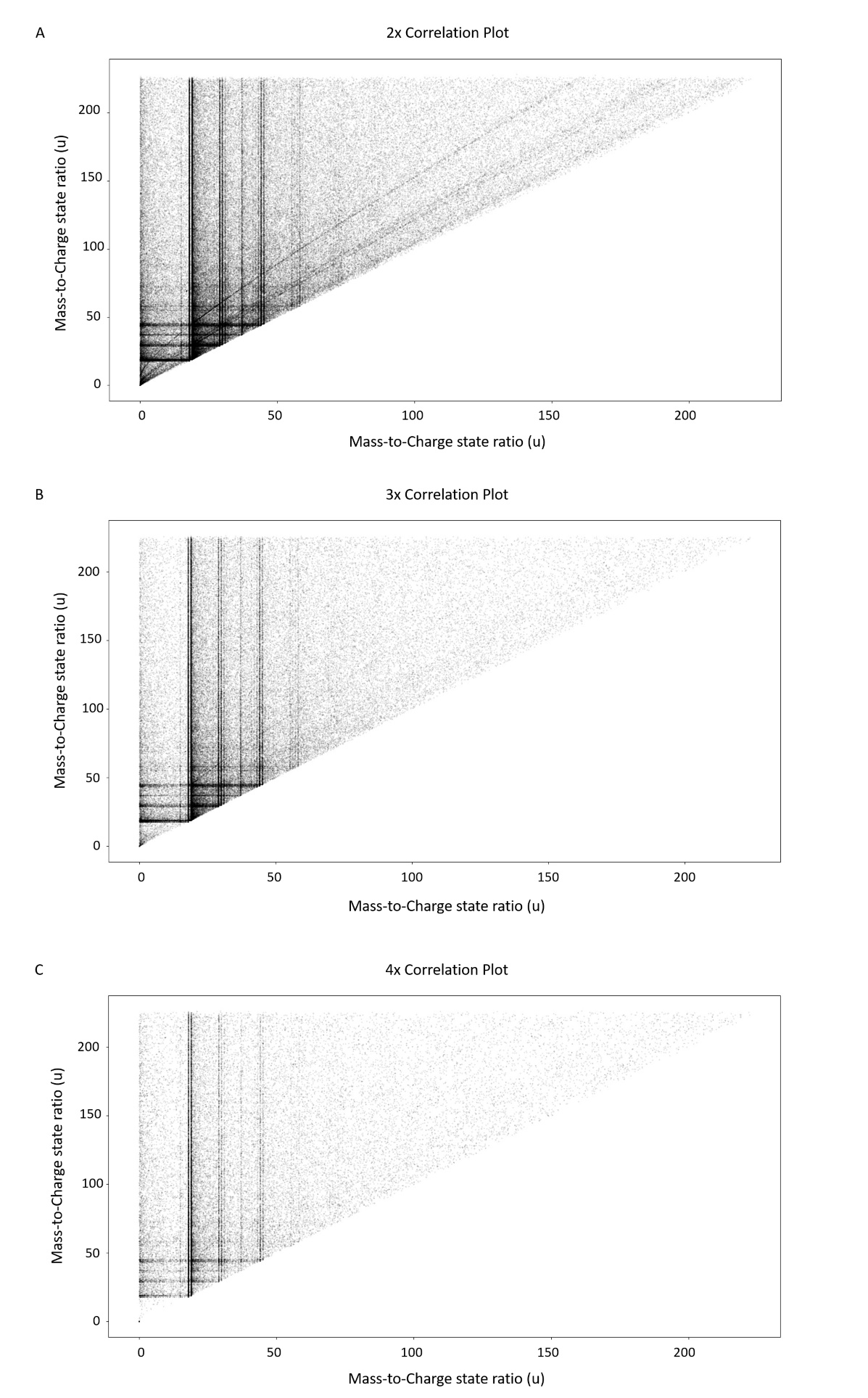


Figure S 4: Correlation histogram for honey for double, triple, and quadruple events. The measured mass-to-charge state ratio m_2_’/q_2_’ of the second event is plotted versus the measured mass-to-charge state ratio m_1_’/q_1_’ of the first event.


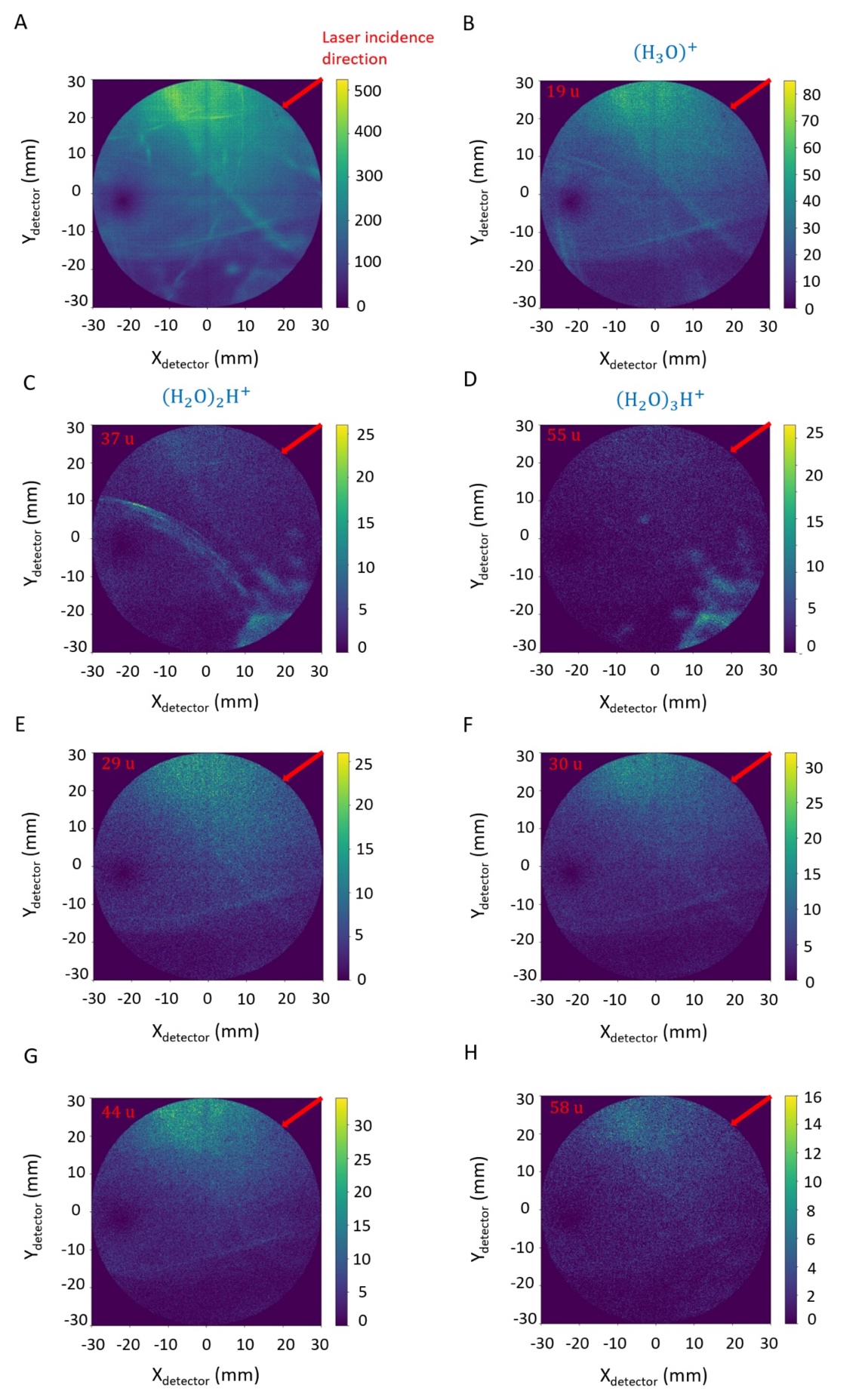


Figure S 5: Desorption maps in (A) all events and (B-D) of different protonated water and in (E-H) for different sugar fragments.
